# Introduction to the Special Issue: “Advance in Recovery and Application of Bioactive Compounds from Seafood”

**DOI:** 10.3390/foods10020266

**Published:** 2021-01-28

**Authors:** Charlotte Jacobsen, Susan L. Holdt

**Affiliations:** Research Group for Bioactives–Analysis and Analysis, National Food Institute, Technical University of Denmark, 2800 Kgs. Lyngby, Denmark; suho@food.dtu.dk

Due to increased focus on a circular bioeconomy, full utilization of marine biomass, including side streams from the seafood processing industry as well as utilization of biomass that has not been used to a great extent in the Western world (e.g., seaweed), is receiving increased attention from both academia and industry. These forms of marine biomass contain a wide range of interesting biomolecules with beneficial health and/or functional properties, which can be exploited for applications in food, feed, dietary supplements or pharma. However, there are several challenges to be overcome. Six high quality research papers about opportunities and challenges in this area of research have been published in this Special Issue. 

Naseri and colleagues developed a multi-extraction process in which protein was extracted from the commercial red seaweed *Euchema denticulatum*, followed by the extraction of carrageenan which is widely used in, for example, the food industry [1]. The process included enzyme assisted aqueous extraction followed by alkaline extraction using N-acetyl-cysteine. Up to almost 60% of the protein in the seaweed could be recovered depending on the enzyme used. 

Seaweed also contains minerals such as iodine. However, in some species the iodine content can be so high that it becomes a concern for the consumer. Nielsen and colleagues studied how the content of iodine can be reduced in the brown seaweed *Saccharina latissima* (sugar kelp) by water blanching or freezing to avoid an excessive intake of iodine via the consumption of this seaweed [2]. They found that the highest reduction in iodine content could be obtained by water blanching at 80 °C for 120 s. They also studied whether water blanching affected the content of other valuable compounds in the seaweed. 

It is well known that fish oil is healthy due to its high content of omega-3 polyunsaturated fatty acids. However, the polyunsaturated nature of fish oil also makes it highly susceptible to lipid oxidation, which leads to formation of undesirable off-flavors. This limits the use of fish oil for food applications. Therefore, strategies to reduce lipid oxidation are necessary. One possible strategy is to emulsify the fish oil in a delivery system which protects the oil against lipid oxidation. This approach requires emulsifiers with good emulsifying properties for preparing emulsions with high physical and oxidative stability. Padial-Dominguez and co-workers optimized the enzymatic hydrolysis conditions used for the preparation of hydrolysates of whey protein and soy protein with optimal emulsifying properties [3]. They also investigated the physical and oxidative stability of fish oil-in-water emulsions prepared with soy protein hydrolysates. They found that soy protein hydrolysates reduced lipid oxidation in such emulsions compared to the use of non-hydrolyzed soy protein isolate. Rahmano-Manglano and colleagues investigated another approach to stabilize fish oil against oxidation, namely, microencapsulation [4]. They studied the influence of the carbohydrate-based wall matrix (glucose syrup or maltodextrin) and the storage temperature (4 or 25 °C) on the oxidative stability of microcapsules, prepared by spray drying emulsions with whey protein hydrolysates as the emulsifier. They found that glucose syrup provided the highest oxidative stability.

Apart from having emulsifying properties, some protein hydrolysates also have antioxidative properties. Tabakaeva and colleagues studied the antiradical properties of hydrolysates and hydrothermal extracts of bivalve mollusks (*Anadara broughtonii*). They also evaluated their effect on lipid oxidation and lipolysis in mayonnaise [5]. They studied hydrolysates obtained either by acid or enzymatic hydrolysis and observed that acid hydrolysates were more efficient than enzymatic hydrolysates in preventing oxidation and lipolysis in mayonnaise. 

Protein hydrolysates may also have bioactive properties, and this was studied in the last paper in this Special Issue. Kang, Skanberg and Myracle studied the anti-hyperglycemic effects including α-glucosidase, α-amylase, and dipeptidyl peptidase-IV (DPP-IV) inhibitory activities and glucagon-like 1 (GLP-1) secretory activity of green crab hydrolysates obtained with different commercially available enzymes [6]. They concluded that hydrolysates obtained by Protamex treatment have the potential for application in type 2 diabetes management.

We hope that this Special Issue will further promote the interest and research in bioactive compounds from seafood. This Special Issue has only scratched the surface, and there is much more to investigate about the possible uses of marine bioactive compounds for feed, food, cosmetics, and pharma; thereby, bringing us closer to an optimized system for the utilization of our marine resources, in order to achieve a sustainable future based on a circular bioeconomy.
